# Mineralogical Composition of the Urinary Stones from Different Provinces in Iraq

**DOI:** 10.1100/tsw.2005.2

**Published:** 2005-01-21

**Authors:** Adnan H. Afaj, Meitham A. Sultan

**Affiliations:** ^1^ Senior Researcher, Head of Environmental Research Center, Ministry of Science and Technology, Iraq; ^2^ Researcher, Environmental Research Center, Ministry of Science and Technology, Iraq

**Keywords:** urinary stones, environmental factors, mineralogy and chemistry of urinary stones, scanning electron microscopy, calcium oxalate stones, phosphate stones, urate stones, Dittmarite

## Abstract

For this study, 25 samples of urinary stones were chosen from different provinces in Iraq as representative sampling localities. These samples of urinary stones were collected to represent kidney, urate, and bladder stones. The main objectives of this study are to try to shed some light on the possibilities of tracking down the effective environmental factors that determine the mineralogical and chemical composition of these stones. The stones were examined using several techniques, the most important of which was the use of the X-ray diffraction (XRD) technique to determine the mineralogical composition of these stones. A scanning electron microscopy (SEM) test was conducted to determine the crystallographic forms and structures for the minerals forming these stones. Optical properties of these minerals were studied using a polarizing microscope. All these techniques revealed that the calcium oxalate, represented in Whewellite mineral, is the most dominant type of these stones, in addition to other minerals such as Hydroxy apatite, Struvite, and Uricite. Dittmarite was pointed out for the first time ever in some samples. This mineral has not been determined in any previous study worldwide.Considering the results of mineralogical and chemical examinations of the urinary stones in question, and the statistical information gathered from the Iraqi Health Ministry, statistical analyses were applied. The ratio of male-female cases in this study happened to be 4:1, which was higher than the ratio in the years 1988—1989 and 1993—1994, 2:1; 3:1 respectively. The highest percentage of the cases was in the 15—50 age group, which is considered as the most productive years of human lifetime. This study showed that one of the most significant factors was that the mineralogical variation of urinary stones in some Iraqi provinces was due to geographical differences, which reflect the variation in lithogenic factors and also climatological factors. Other factors may be socioeconomic, genetic, physiological, and pathological, which remain the important factors in forming urinary stones.

## INTRODUCTION

Urinary stones have afflicted humankind since antiquity. There is evidence of urinary stones as early as 7000 years ago and perhaps earlier. The earliest recorded example is bladder and kidney stones detected in Egyptian mummies dating to 4800 years B.C. (a urinary stone belonging to a 16-year-old boy). A urinary stone was found in a boy from 3000 years ago in America[1], however, the oldest surgical operation of removing a urinary stone was referenced in the Indian literature from 500–1000 years B.C.

Socioeconomic, geographic, and genetic factors play important roles in determining the prevalence and composition of urinary stones in different parts of the world. A number of papers have previously reported the high prevalence and particular patterns of stone disease in developing countries.

In general, urinary stones may contain various combinations of chemicals. The most typical stones contain calcium in combination with either oxalate or phosphate. Much less common are the uric acid stones and the rare cystine stones. Urinary stones have become increasingly common in most parts of the world in recent years[2]. It was found that 80% of kidney stone cases are among men and only 20% are women.

The occurrence and composition of urinary stones show geographical differences[3]. These differences reflect variation in lithogenic factors and climatological factors. These differences can be seen in kidney stone belts around the world, as in the southeastern area of the U.S., where North Carolina has more kidney stone cases per capita than other states. Several factors probably come into play to create this situation. One is the typical southern diet that is high in green vegetables and brewed tea, both of which are high in oxalates. The second is the hot climate, which causes increased amounts of perspiration and loss of body fluid.

It was also found that the incidence, composition, and clinical characteristic of urinary stones in children vary greatly according to the geographic region[4] and they remain a major source of morbidity in countries that constitute the stone belt from Turkey and Egypt in the West to Indonesia in the East[5]. The etiology also varies according to geographic areas. Bladder stones have been noted to be endemic in developing countries such as those in Southeast Asia. The high prevalence of these stones is attributed to the predominantly cereal-based, low protein diet and high ambient temperature. In contrast, in European and North American countries, kidney stones predominate. Recent studies from South Africa and Iran indicate that incidence of renal stones become more frequent as these countries become more affluent[5].

In the Arabian Gulf states, calculous disease of the urinary tract is common[6], but reports on the disease are scarce. Several studies have been done to examine urinary and kidney stones[7,8,9,10,11,12].

## MATERIALS AND METHODS

In 1997, 25 urinary stones from different patients were collected from Iraqi hospitals in some provinces of Iraq (Table 1); the stones were removed by surgical operations. The patients were 20 males and 5 females of ages ranging from 16–85 years. Male to female ratio was about 4:1. The types of urinary stones that were collected were 12 bladder stones, 3 urate stones, and 10 kidney stones (Table 2).

The samples were prepared for analysis following the procedure recommended by Lin et al.[13]. At first, the urinary stones were washed with distilled water to remove possible contamination and then dried at 100°C for about 3–4 h.

**TABLE 1**
**Distribution of Urinary Stone Samples According to Provinces, Sex, and Age**

| No. | City | No. of Samples | Males | Females | Age (Year) |
|---|---|---|---|---|---|
| 1 | Mosul | 8 | 5 | 3 | 20–65 |
| 2 | Baghdad | 12 | 10 | 2 | 16–85 |
| 3 | Babel | 1 | 1 | | 45 |
| 4 | Najaf | 1 | 1 | | 16 |
| 5 | Qadysia | 1 | 1 | | 35 |
| 6 | Basrah | 2 | 2 | | 80 |
| | Total | 25 | 20 | 5 | 16–85 |

**TABLE 2**
**Number and Types of Urinary Stones**

| Type of Stone | No. | % |
|---|---|---|
| Kidney | 10 | 40 |
| Urate | 3 | 12 |
| Bladder | 12 | 48 |
| Total | 25 | 100 |

Stone analysis is based on chemical and physical methods. It may be emphasized that no single method or technique provides total information about the structure and composition of these stones. At least two different methods have to be combined for an accurate analysis of stone[14]. The composition, chemistry, morphology, and structure of urinary stones were studied by several techniques. The mineralogical study included the analysis of 25 samples by X-ray diffraction (XRD) using a Philips PW5 1337 automatic X-ray powder diffractometer; this analysis was supported by the analysis of 8 samples by infrared spectroscopy (IR) to identify the composition and crystalline or noncrystalline components of the studied samples using SP3-300. Ten samples were studied using scanning electron microscopy (SEM), model JEOL-6400, in order to determine the nature of crystalline texture and microstructure of the stones and five samples were studied by polarizing microscopy (PM), Leitz Model, to identify the optical properties of the minerals constituent and crystallinity of the studied urinary stones.

The physical properties, surface shape, and cross-section were examined with a magnification glass lens (×10).

## RESULTS AND DISCUSSION

### Mineralogical Analysis

Knowledge of the composition of urinary stone is an important, and sometimes the only, indication of the disorder, therefore it must be known[15]. Stone components may be mineral, organic, or both. The same chemical components may crystallize in different forms; for instance, calcium oxalate may be present in the form of calcium oxalate monohydrate (COM), dihydrate (COD), or trihydrate (COT), therefore proper stone analysis has to identify not only the molecular species, but also the crystalline form within the chemical constituents[14] (Table 3).

**TABLE 3**
**Crystalline Components**

| Component | Formula | Mineralogical Name |
|---|---|---|
| COM | $Ca_2O_4.H_2O$ | Whewellite |
| COD | $Ca_2O_4.2H_2O$ | Weddellite |
| Magnesium ammonium phosphate hexahydrate | $MgNH_4PO_4.6H_2O$ | Struvite |
| Magnesium ammonium phosphate monohydrate | $MgNH_4PO_4.H_2O$ | Dittmarite |
| Calcium hydrogen phosphate dihydrate | $CaHPO_4.2H_2O$ | Brushite |
| Carbonate-apatite | $Ca_{10}(PO_4CO_3)_6(OH)_8$ | Carbonate-apatite |
| Hydroxyl-apatite | $Ca_{10}(PO_4)_6(OH)_2$ | Hydroxyl-apatite |
| Anhydrous tricalcium phosphate | $Ca_3(PO_4)_2$ | Whitlockite |
| Magnesium diabasic phosphate trihydrate | $MgHPO_4.3H_2O$ | Newberyite |
| Uric acid | $C_5H_4N_4O_3$ | Uric acid |
| Uric acid dihydrate | $C_5H_4N_4O_3.2H_2O$ | Uric acid dihydrate |
| Sodium acid urate | $NaC_5H_3N_4O_3.H_2O$ | Sodium acid urate |
| Ammonium acid urate | $NH_4C_5H_3N_4O_3.H_2O$ | Ammonium acid urate |
| Cystine | $C_6H_{12}N_2O_4S_2$ | Cystine |
| Xanthine | $C_5H_4N_4O_2$ | Xanthine |

**TABLE 4**
**Composition of Urinary Stone Samples Analyzed by XRD**

| Type of Stone | No. | % |
|---|---|---|
| Calcium oxalate | 11 | 46.1 |
| Phosphate | 10 | 38.4 |
| Urate | 4 | 15.4 |

Because almost all constituents of urinary stones are inorganic minerals or organic crystalline compounds, urinary stone can be analyzed with the analytical methods used in mineralogy[15, 16, 17, and 18].

XRD analysis of 25 samples of urinary stones revealed three main crystalline groups (Table 4). Of the 25 urinary stones analyzed by the XRD technique, 11 stones belonged to the calcium oxalate group (COM and COD) representing 46.1% of total samples in the form of Whewellite and Weddellite minerals, 10 stones belonged to the phosphate group representing 38.4% of total samples in the form of Dittmarite and Struvite minerals and Hydroxy apatite, and 4 stones belonged to the urate group representing 15.4% of total samples in the form of uric acid (Uricite). Seven samples were analyzed using the IR technique and were classified into two groups (Table 5). These groups are calcium oxalate stones in the form of Whewellite and Weddellite minerals and phosphate stones in the form of Dittmarite, Struvite, and Hydroxy apatite minerals.

## Morphology and Structure

Superficial examination and cross-section of stones depend not only on molecular composition and on crystalline form, but on anatomical conditions and metabolic activity of stone disease.

**TABLE 5**
**Composition of Urinary Stone Samples Analyzed by IR**

| Type of Stone | No. | % |
|---|---|---|
| Calcium oxalate | 4 | 57.1 |
| Phosphate | 3 | 42.8 |

Color, texture, structural features, shape, and size of crystals are significant features that give information on the age of the stone and possible processes of crystallization. For instance, dark-colored Whewellite stone can be usually considered an old inactive stone with a slow growth rate that allowed the incorporation of high quantities of urinary pigments in the inner and superficial layers. By contrast, a very pale-colored Whewellite structure is the evidence for crystalline growth and very active lithogenic process[14](researchers tried to establish relationships between composition and structure of urinary stone by observations of both surface and cross-section using dissecting microscope). The physical properties, surface shape, and cross-section were examined with a magnification glass lens (×10) for eight samples of urinary stones. The description of studied samples is summarized as follows:

1. Calcium oxalate stone: The studied samples of these stones were kidney and bladder stones. The kidney stone sample had a spherical shape 1 cm in diameter and had many elongated nodes on its surface. It represented in Whewellite mineral (Fig. 1). The bladder stone was oval in shape with very clear concentric layers like an onion and was 6 cm in diameter. It was brownish in color (Fig. 2).
2. Phosphate stone: The studied samples were urate and bladder stones. The surface of these stones seemed to be heterogeneous, smooth, rough, or chalky. The urate stone, which represented in Dittmarite mineral, had more or less cardiac or staghorn shape with smooth surface, and was 3 cm long. Its color was white to brownish gray (Fig. 3). The other stones, which represented in Struvite and Hydroxy apatite minerals, were rounded in shape and 2 cm in diameter, very rough surface, and brownish in color (Fig. 4), or may be irregular in shape and also brownish in color (Fig. 5).
3. Urate stone: The studied samples of these stones were kidney stones and they have different sizes with irregular and smooth to rough surfaces. The colors were beige to brown. They represented in Uricite mineral (Fig. 6).

## SEM

SEM allows a morphological analysis of the stone surface and gives information about the nature of crystalline compounds, shape of the crystal, internal structure, location of component, and some data about relations between crystals and matrix. Ten samples were examined with the SEM technique.

1. Calcium oxalate stones: This type represented in Whewellite mineral. It crystallizes in monoclinic system. Whewellite takes different forms as flaky, bladed, and elongated columnar crystals[9,12] (Fig. 7).
2. Phosphate stones: This type represented in:

- Dittmarite: It crystallizes in orthorhombic system. It takes a spherical bladed or flaky spheroid form, and also bladed and disc form[9,12] (Fig. 8).
- Struvite: It crystallizes in orthorhombic system. Its crystals take the bladed and prismatic form[9,12] (Fig. 9).

3. Urate stones: This type represented in uric acid (Uricite). It crystallizes in monoclinic system; it is polymorphic, it takes a radial, fibular and equate, prismatic crystal form[9,12,16] (Fig. 10).

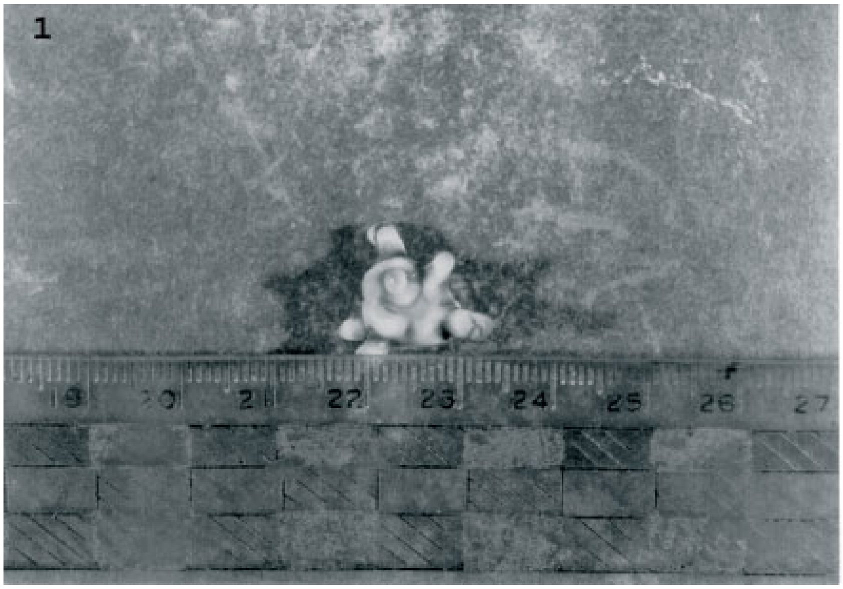

**FIGURE 1.** Calcium oxalate kidney stone. See text for description.

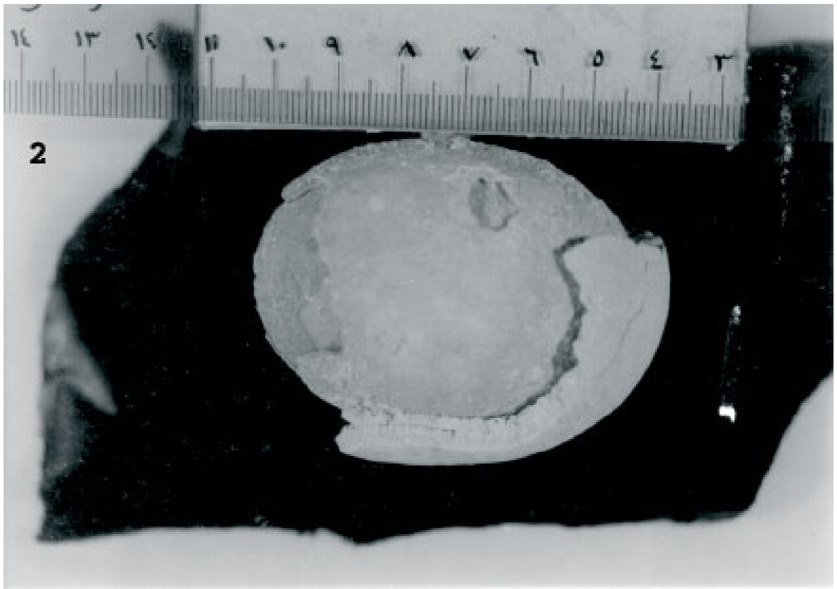

**FIGURE 2.** Calcium oxalate bladder stone. Represented in Whewellite mineral.

## PM

The samples of urinary stones were studied by PM in order to identify the optical properties of the minerals constituent and crystallinity of the studied urinary stones[19,20,21].

Thin section studies of urinary stones using PM showed that the calcium oxalate stones represented in Whewellite minerals were brownish to light brown in color, and showed also concentric and radial patterns with organic matter bands (Fig. 11). The phosphate stones, that represented in Dittmarite mineral, were light to dark brown to brownish, and showed a very clear concentric radial pattern with dark brown color. Very thin organic concentric bands also may be seen.

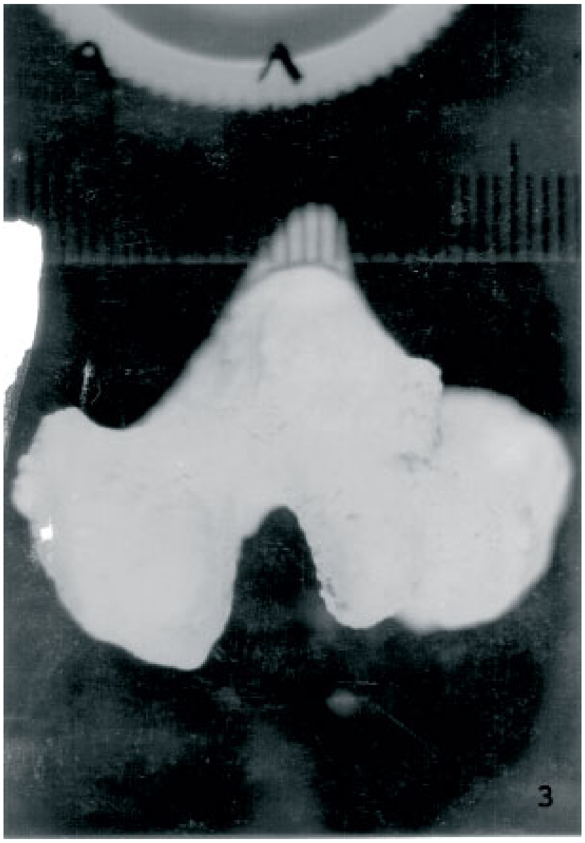

**FIGURE 3.** Phosphate urate stone. See text for description.

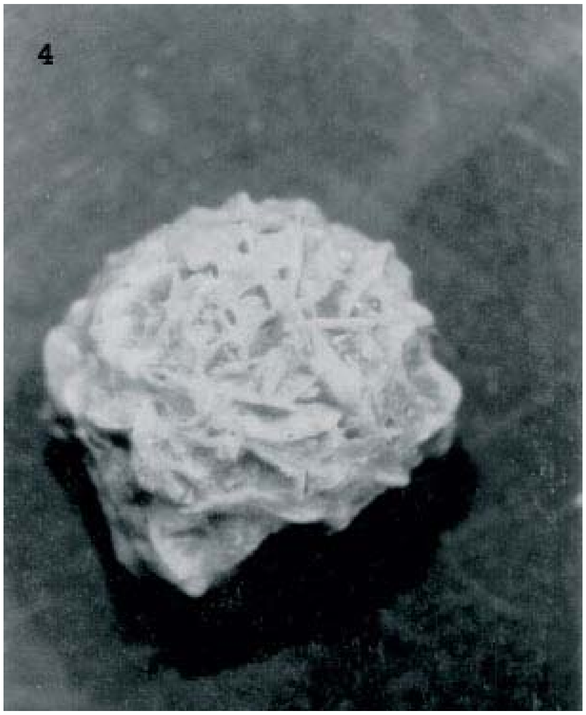

**FIGURE 4.** Phosphate urate stone. See text for description.

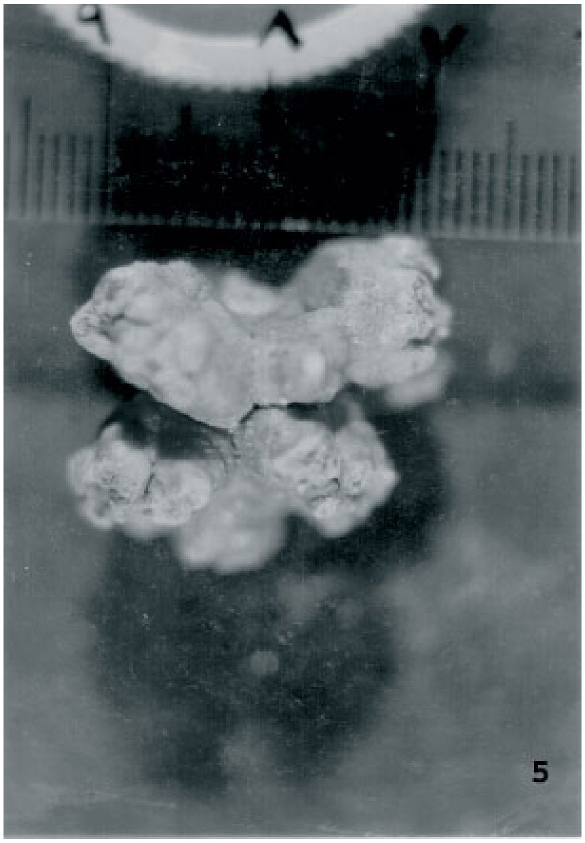

**FIGURE 5.** Phosphate urate stone. See text for description.

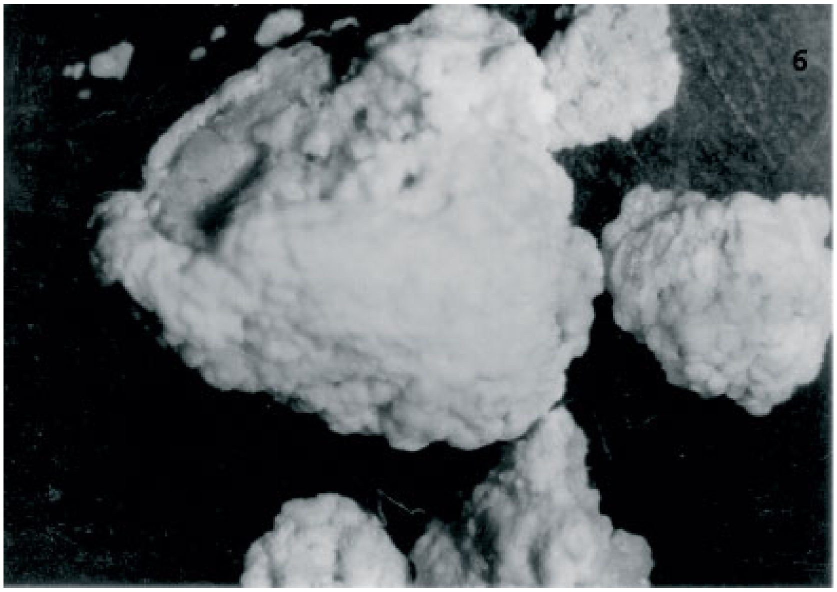

**FIGURE 6.** Urate kidney stones with different shapes and sizes. See text for description.

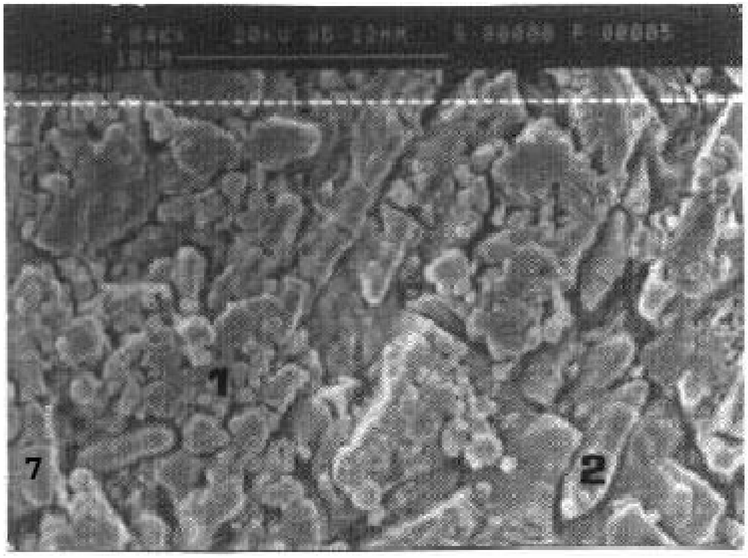

**FIGURE 7**. SEM photo of Whewellite mineral (monoclinic system), which shows (1) aggregation or (2) columnar shapes.

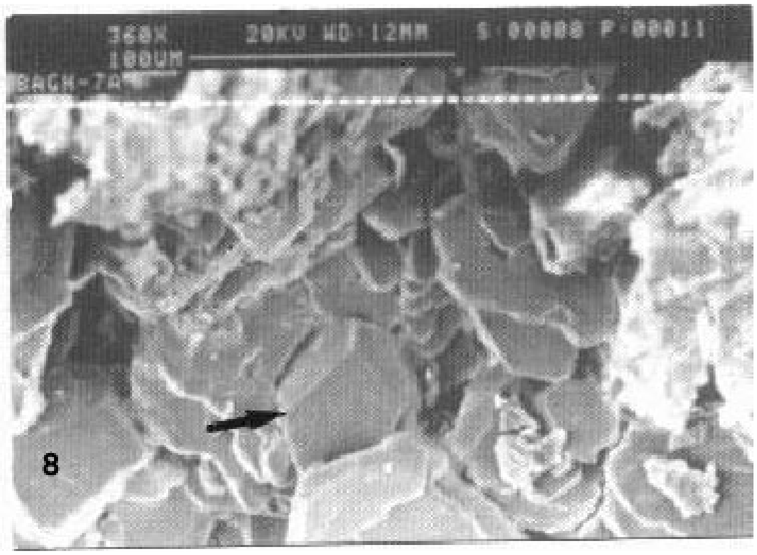

**FIGURE 8.** SEM photo of Dittmarite mineral (orthorhombic system), which shows spherical bladed or bladed and disc shapes.

## CONCLUSION

The study of 25 urinary stones of Iraqi patients from different provinces of Iraq, ranging in age from 16–85 years, showed a mean range of 35 years and a male to female ratio 4:1.

The combined results of XRD and IR analysis of these urinary stones showed that the most common type of urinary stone in Iraq was calcium oxalate stone (in the form of Whewellite) representing 46.1% of the total samples, followed by phosphate stone (in the form of Dittmarite, Struvite, and Hydroxy apatite) representing 38.4% of the total samples, and urate stone (in the form of Uricite) representing 15.4% of the total samples (Table 4).

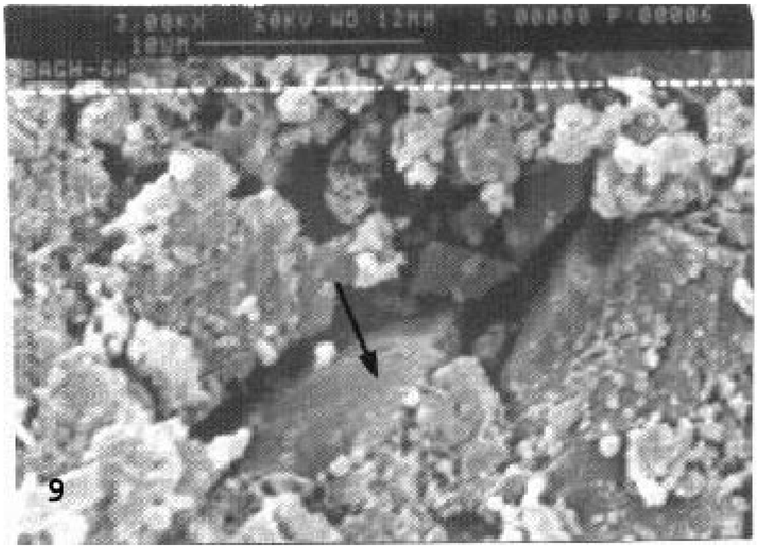

**FIGURE 9.** SEM photo of Struvite mineral (orthorhombic system), which shows (1) flaky prismatic shape with sharp edges, (2) radial texture grown on or between the flaky prismatic forms, (3) honeycomb-like surface of the mineral Hydroxy apatite.

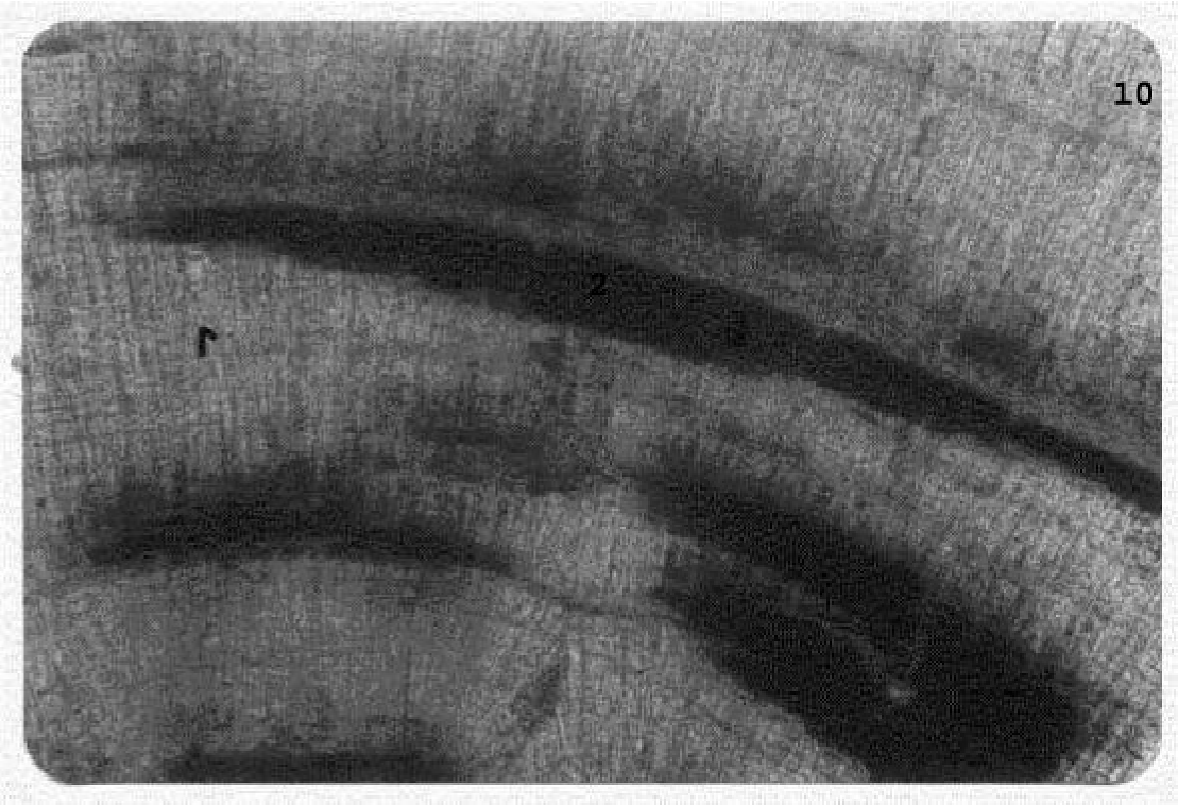

**FIGURE 10.** SEM photo of Uricite mineral (monoclinic system), dendritic and rather irregular crystals, with well crystalline face of prismatic crystal (arrow).

**TABLE 6**
**Comparison of Urinary Stone Type Between Baghdad and Mosul Provinces**

| Type of Urinary Stone | Baghdad Province | Mosul Province |
|---|---|---|
| Calcium oxalate | 18.7% | 25.% |
| Phosphate | 43.7% | 12.5% |
| Urate | 12.5% | 6.3% |

In order to determine and also to indicate some of the environmental factors that may affect the variability of the urinary stone in some provinces of Iraq, a comparison was undertaken between the type of the urinary stones in Mosul province, which is located in northern Iraq in the foothill zone, with the same type of urinary stone in Baghdad province, which is located in the Mesopotamian region. The high incidence of calcium oxalate stones in the patients from Mosul province may be attributed to the high percentage of calcium within the Gypseous soil in Mosul province compared to the silty soil poor in calcium in Baghdad province. The high incidence of phosphate stones in Baghdad province in comparison with the same stones in Mosul province may be due to a high concentration of magnesium in Tigris River water and in the soil of Baghdad province (Table 6).

Worldwide comparisons have been undertaken that show agreement that calcium oxalate stones are the most common of all the studied stones, followed by uric acid stones (Table 7). Phosphate stones (representing all minerals that contain phosphate in their chemical composition) showed low incidence percentages in Iraqi patients (38.4%), in comparison with the incidences in the U.S. and the world overall,

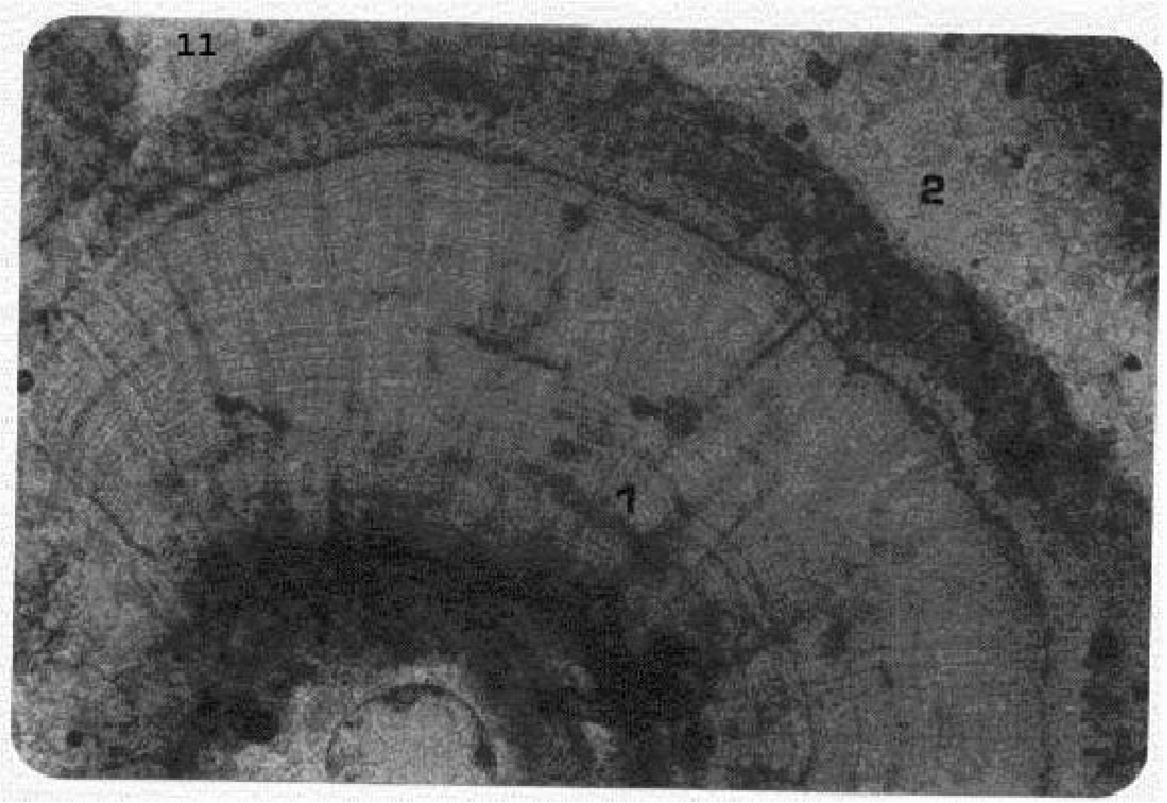

**FIGURE 11.** PM photo showing the (1) Dittmarite mineral with radial and concentric structures, with very thin black layer, represented in the organic matter, (2) with aggregation of light crystal represented in Whewellite. ×200.

but it is still higher in comparison with the incidences in the Arabian Gulf. The percentage of uric acid stones in Iraqi patients is higher than the percentage of this type of stone in American and world patients, while cystine stones are rarely found in Iraqi patients compared with Americans and world patients (Table 7).

In general, this study showed that:

1. The most common type of urinary stone in Iraq was calcium oxalate (in the form of Whewellite) representing 46.1% of the total samples, followed by phosphate (in the form of Dittmarite, Struvite, and Hydroxy apatite) representing 38.4% of the total samples, and urate (in the form of Uricite) representing 15.4% of the total samples.
2. The most significant factor, which affects the mineralogical variation of urinary stones in some Iraqi provinces, was the geographical differences reflecting the variation in lithogenic factors and maybe also climatological factors. Other factors may be socioeconomic, genetic, physiological, and pathological, which remain the important factors in forming urinary stones.

3. Dittmarite was pointed out for the first time ever in some samples. This mineral has not been determined in any previous study in the Gulf region.

**TABLE 7**
**Comparison of Stone Type Percentages Between Iraqi, Arabian Gulf, U.S., and World Patients**

| Type of Stone | World | U.S. | Arabian Gulf State* | Iraq (Present Study) |
|---|---|---|---|---|
| Calcium oxalate | 30 | 32 | 38.5 | 46.1 |
| Phosphate | 60** | 62.5** | 11.5** | 38.4** |
| Uric acid | 5 | Rare | 11.5 | 15.4 |
| Cystine | 2 | 3 | — | Rare |

* There is a fourth group of urinary stones that are a mixture of group 1 and group 2 (mixed stones) and represent 38.5%.

** The percentage of the phosphate stones is the sum of all stones that contain phosphatic minerals.

## ACKNOWLEDGMENTS

I would like to express my sincere gratitude and deep appreciation to the German Academic Exchange Service for the grant and the invitation to Germany. My deep thanks and gratitude to Prof. Dr. H. Ehses, Director of the Geological Survey and Mining Authority of Rhineland Pfalz, for his help and kindness to make this invitation possible through the preparation of workplace and all other important materials. My deepest thanks goes to my best friend and colleague, Dr. Peter Schäfer, for his help, encouragement, and generous advice to make this grant and this work possible. Finally, I would like to express my deep thanks for all workers in the Geological Survey and Mining Authority of Rhineland Pfalz for their help, especially Mr. R. Müller, Ms. A. Schauß, and Mr. S. Leonhardt.

## BIOSKETCH

**Adnan H. Afaj, Ph.D**., is Head of the Environmental Research Center/Ministry of Science and Technology in Baghdad, Iraq. Dr. Afaj received his B.Sc. in geology from the University of Baghdad in 1974, a Diploma in Carbonate Sedimentology from the Johannes Gutenberg University, Mainz, West Germany in 1980, and his Ph.D. in Environmental Geochemistry also from the Johannes Gutenberg University in 1983.

From 1984–1986, Dr. Afaj served as Geologist in the Geological Survey and Mining/Ministry of Industry and Minerals in Baghdad; from 1986–1991, Lecturer in the University of Basrah, Iraq; from 1991–1992, Assistance Professor at the University of Basrah; from 1992–2003, Senior Researcher in the Environmental Research Center, Baghdad; and from 2003 until now, Senior Researcher and Head of Environmental Research Center in the Ministry of Science and Technology.

Dr. Afaj's professional interests include stable isotope geochemistry, medical geochemistry, surface and groundwater pollution, geochemistry of carbonate rocks, geochemistry of recent sediments, air pollution, and GIS techniques in environmental studies. Most of his research work focuses on environmental geochemical studies in the field of surface and groundwater pollution, especially in the Tigris and Euphrates Rivers and in some groundwater aquifers in South and East Iraq. Using bioindicators in surface water is one of the important goals in these environmental studies. Medical geological studies have importance in these environmental studies, especially in the field of urinary stones and also in the prediction of cancer disease using trace metals in human body.

Dr. Afaj received the following grants: (1) DAAD – Scholarship 2000 (4 months) and (2) DAAD – Scholarship 2004 (2 months).

**Meitham A. Sultan, MSc**. in Geochemistry, is a researcher in the Environmental Research Center/Ministry of Science and Technology in Baghdad, Iraq. Mr. Sultan received his B.Sc., in geology from the University of Baghdad in 1993, M.Sc. in Medical geochemistry from University of Baghdad in 1998. Now he is Ph.D. student in Baghdad University.

From 1994-2003, Mr. Sultan served as geologist in Environmental Research Center in Baghdad. From 2003 until now Mr. Sultan is a researcher in Environmental Research Center, Ministry of Science and Technology.

Mr. Sultan's professional interests include environmental geochemistry and medical geochemistry, and GIS technique. Most of his work focuses on environmental geochemistry, medical geochemistry and GIS technique in environmental studies.
